# Giant Merkel Cell Carcinoma Masquerading as a Benign Cyst on the Buttock of an African American Man

**DOI:** 10.1155/2011/849767

**Published:** 2011-09-22

**Authors:** Marissa J. Perman, Janelle M. King, Laurel L. Leithauser, Hugh M. Gloster

**Affiliations:** Department of Dermatology, College of Medicine, University of Cincinnati, Cincinnati, OH 45267, USA

## Abstract

We report a case of a 60-year-old African American man who presented with a 4-year history of a previously asymptomatic, recently enlarging nodule on his left buttock, which was initially presumed to be an epidermoid cyst. Physical examination revealed a large, fixed, subcutaneous tumor, and a biopsy revealed merkel cell carcinoma. Immunohistochemical staining was positive for pankeratin, CAM 5.2, synaptophysin, and CD56 and negative for CK7, CK20, TTF-1, chromogranin, CD3, CD20, CD57, MART1, and HMB 45. The patient underwent wide local excision of the lesion with removal of the fascia overlying the gluteus and full body positron emission tomography (PET) and was found to have Stage IIb disease. He subsequently received adjuvant radiotherapy limited to the tumor bed at a dose of 60 gray.

## 1. A Case Report

**Figure 1:**
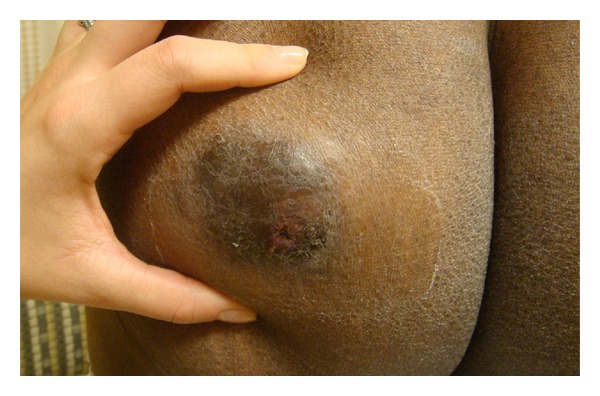
A 9 by 10 centimeter fixed, subcutaneous tumor with overlying hyperpigmentation and slight scale.

A 60-year-old African American male presented with a 4-year history of an asymptomatic stable nodule involving the left buttock. The lesion was initially thought to be an epidermoid cyst that did not require treatment. However, he returned after the nodule rapidly increased in size over 2-3 months. Physical examination revealed a 9 by 10 centimeter fixed, subcutaneous tumor with overlying hyperpigmentation and slight scale (Figure 1).

**Figure 2:**
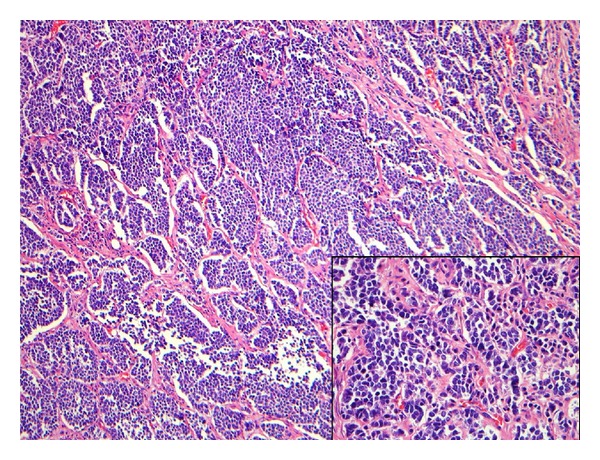
Ribbons of small, round, uniform blue cells with nuclear molding (hematoxylin-eosin, original magnification ×100, inset: original magnification ×400).

A punch biopsy specimen demonstrated merkel cell carcinoma (MCC) (Figure 2). Immunohistochemistry stains were positive for pankeratin, CAM 5.2, synaptophysin, and CD56 and negative for CK7, CK20, TTF-1, chromogranin, CD3, CD20, CD57, MART1, and HMB 45. Full body positron emission tomography (PET) demonstrated uptake at the sight of the tumor and a local inguinal node. The patient underwent wide local excision of the mass with removal of the fascia overlying the gluteus. The underlying stripped muscle was grossly negative. The abnormal node on PET was identified as the sentinel lymph node and was negative, both by fine needle aspiration and excision. Upon pathologic review, the tumor involved only the deep margin. Therefore, he was diagnosed with Stage IIb MCC (pT3N0M0). Given the positive deep margin, the patient subsequently received adjuvant radiotherapy limited to the tumor bed at a dose of 60 gray. Adjuvant chemotherapy was not indicated per National Comprehensive Cancer Network guidelines [1].

## 2. Discussion

MCC is an aggressive skin cancer usually noted on sun-exposed sites of elderly Caucasian men. The disease-associated mortality ranges from 33 to 50% [2, 3]. Merkel cells were initially believed to originate from neural crest-derived neuroendocrine cells; however, they are now thought to be derived from pluripotent epidermal stem cells [4]. Most MCC are likely induced by the merkel cell polyomavirus [3]. CK20 is the most frequently utilized immunohistochemical stain used to diagnose MCC and is positive in 89–100% of cases [1].

Key clinical characteristics of MCC include patient ethnicity, tumor location, and rate of tumor growth. The vast majority of patients (~98%) with MCC are white [2, 4, 5]. The most common tumor location involves the head and neck in sun-exposed regions. In addition, rapid rate of expansion (≤3 months) is often associated with MCC [2].

Immunosuppression is also a risk factor for MCC. Specifically, patients with human immunodeficiency virus, chronic lymphocytic leukemia, and solid organ transplantation are at increased risk for MCC [2].

We report an unusual presentation of MCC initially thought to be a benign slow growing cyst of the buttock in an otherwise healthy 60-year-old African American male. We highlight this case because of the patient's ethnicity, the length of time the tumor was present (four years) before the rapid growth phase, the non-sun-exposed primary site, and lack of immunosuppression. Furthermore, the tumor was relatively large (9 cm) and was negative for the CK 20 marker.

We aim to make dermatologists more aware of this unique presentation of a slow-growing giant MCC in a non-sun-exposed site of an African American patient.
